# The effects of creatine supplementation on cognitive performance—a randomised controlled study

**DOI:** 10.1186/s12916-023-03146-5

**Published:** 2023-11-15

**Authors:** Julia Fabienne Sandkühler, Xenia Kersting, Annika Faust, Eva Kathrin Königs, George Altman, Ulrich Ettinger, Silke Lux, Alexandra Philipsen, Helge Müller, Jan Brauner

**Affiliations:** 1 Department of Psychology, University of Bonn, Kaiser-Karl-Ring 9, 53111 Bonn, Germany; 2 Department of Psychiatry and Psychotherapy, University Hospital Bonn, Bonn, Germany; 3 Department of Psychiatry and Psychotherapy, University Medical Center, Mainz, Germany; 4 Manchester University NHS Foundation Trust, Manchester, UK; 5 Department of Health, Witten/Herdecke University, Witten, Germany; 6 Department of Computer Science, University of Oxford, Oxford, UK; 7 Future of Humanity Institute, University of Oxford, Oxford, UK

**Keywords:** Creatine, Cognition, Intelligence, Cognitive performance, Raven’s Advanced Progressive Matrices, Backward Digit Span, Working memory, Deductive reasoning, Randomised controlled trial, RCT

## Abstract

**Background:**

Creatine is an organic compound that facilitates the recycling of energy-providing adenosine triphosphate (ATP) in muscle and brain tissue. It is a safe, well-studied supplement for strength training. Previous studies have shown that supplementation increases brain creatine levels, which might increase cognitive performance. The results of studies that have tested cognitive performance differ greatly, possibly due to different populations, supplementation regimens, and cognitive tasks. This is the largest study on the effect of creatine supplementation on cognitive performance to date.

**Methods:**

Our trial was preregistered, cross-over, double-blind, placebo-controlled, and randomised, with daily supplementation of 5 g for 6 weeks each. We tested participants on Raven’s Advanced Progressive Matrices (RAPM) and on the Backward Digit Span (BDS). In addition, we included eight exploratory cognitive tests. About half of our 123 participants were vegetarians and half were omnivores.

**Results:**

Bayesian evidence supported a small beneficial effect of creatine. The creatine effect bordered significance for BDS (*p* = 0.064, *η*^2^_P_ = 0.029) but not RAPM (*p* = 0.327, *η*^2^_P_ = 0.008). There was no indication that creatine improved the performance of our exploratory cognitive tasks. Side effects were reported significantly more often for creatine than for placebo supplementation (*p* = 0.002, RR = 4.25). Vegetarians did not benefit more from creatine than omnivores.

**Conclusions:**

Our study, in combination with the literature, implies that creatine might have a small beneficial effect. Larger studies are needed to confirm or rule out this effect. Given the safety and broad availability of creatine, this is well worth investigating; a small effect could have large benefits when scaled over time and over many people.

**Trial registration:**

The trial was prospectively registered (drks.de identifier: DRKS00017250, https://osf.io/xpwkc/).

**Supplementary Information:**

The online version contains supplementary material available at 10.1186/s12916-023-03146-5.

## Background

Given the important role cognition plays in daily life, enhancing cognition safely and cheaply is highly desirable. Creatine is safe, well-tolerated, and cheap [1]. Strength athletes have benefited from creatine supplementation for over 30 years [2, 3]. Slight weight gain due to water retention is the only consistently reported side effect [1, 4–6].

While the safety and athletic benefits of creatine are well established, its potential cognitive benefits are still unclear. A systematic review tentatively suggests that creatine supplementation may improve “short-term memory”/working memory and “intelligence/reasoning” in healthy individuals [7]. The few studies that have tested this have had heterogeneous results, but they have also used very different populations (such as vegetarians, omnivores, varying age groups), supplementation doses and durations, and cognitive tasks (including different kinds of memory, reaction time, reasoning, inhibitory control, attention, and task switching). The study with the largest effect, Rae et al. [8], tested the effect of creatine supplementation in 45 young vegetarian adults on working memory and abstract reasoning using the Backwards Digit Span (BDS) and Raven’s Advanced Progressive Matrices (RAPM), respectively. Their study was placebo-controlled, randomised, and double-blind. Rae et al. [8] found creatine supplementation had a large and highly significant (i.e.* p* < 0.001) positive effect on both tasks. We deemed this study particularly worth replicating.

Supplementing creatine may benefit cognition as muscle and brain cells use creatine to access more energy when demand is high. They store creatine as phosphocreatine, which acts to regenerate the energy-providing adenosine triphosphate (ATP) [9, 10]. The energy demand of neurons can increase rapidly; maintaining ATP concentration despite increased demand may explain the potential effect of creatine intake on cognition [11]. The crucial role of creatine in brain metabolism is supported by evidence from Cerebral Creatine Deficiency Syndromes. Conditions causing brain creatine deficiency result in profound intellectual disability which can be reversed by creatine supplementation [12].

Dietary creatine is primarily contained in meat, fish, and a small amount in some dairy products [13, 14]. However, typical supplementation doses of creatine (5 g per day) are equivalent to more than 1 kg of meat consumption per day [13], which is substantially higher than the combined dietary intake and synthesis in most people [13]. So, one might expect creatine supplementation to make a difference despite creatine being produced by the body and being present in common foods.

Creatine intake increases the level of creatine in the blood serum [15, 16]. Crucially, Dechent et al. [17] found brain creatine increased by 8.7% following a 20 g/day 4-week supplementation regime; two further studies have confirmed varying supplementation regimes can increase brain creatine [18, 19] (however, see [20–22]).

It is unclear if creatine supplementation has similar effects on omnivores and vegetarians. Rae et al. [8] only included vegetarians. Another study comparing memory improvement under creatine supplementation in omnivores and vegetarians found that creatine supplementation benefited memory only for vegetarians but not omnivores [23]. Vegetarians have been found to have lower serum and muscle creatine concentration, but comparable total brain creatine to omnivores [22, 24, 25]. In this study, we included both omnivores and vegetarians to allow comparison. We hypothesised that creatine supplementation would improve working memory and reasoning ability in vegetarians. We also hypothesised that the improvement would be greater in vegetarians than in omnivores.

To test these hypotheses, we approximately replicated the study design and treatment (5 g per day of creatine for 6 weeks) used by Rae et al. [8]. We included the same primary outcome measures, the Backwards Digit Span and 10-min standardised subtests of Raven’s Advanced Progressive Matrices. In addition, to investigate a broader range of cognitive functions, we included exploratory tests on attention, verbal fluency, task switching, and memory.

## Methods

### Trial design

**Fig. 1:**
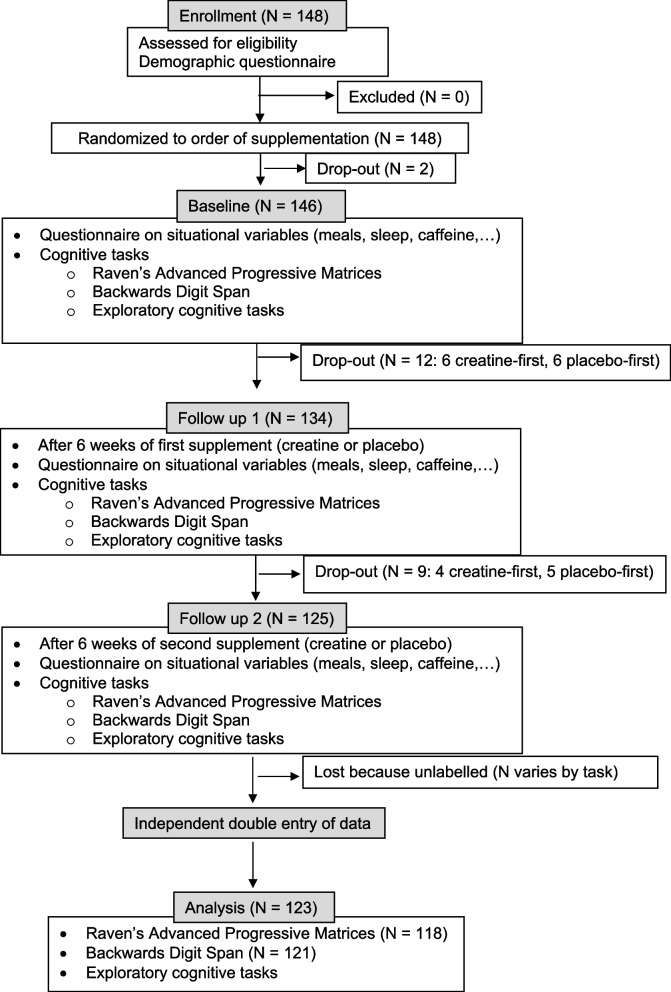
Participant flow through the study

We conducted a randomised, placebo-controlled, double-blind, cross-over study. The primary endpoints are the scores in the cognitive tasks after 6 weeks of each supplementation. Six weeks is the duration used by Rae et al. [8]. We learned from private correspondence with Turner et al. [26] that they chose a liberal 5-week washout period for their brain creatine study based on the muscle creatine literature, which suggests 5 weeks is sufficient [27–29]. Given the way creatine is transported, stored, and excreted, they expected the same washout period to be sufficient for the brain. They confirmed that in their study, brain creatine levels were back to normal after the washout. This is evidence that 5 weeks is an upper bound for how long brain creatine takes to wash out. We also see no reason why brain creatine would take longer to wash out than muscle creatine. Note that Turner et al. [26] used a shorter (7 days) but higher-dosed (20 g) supplementation regimen compared to the present study. However, there is evidence that the supplementation regimens are equivalent in terms of creatine saturation (e.g. [27]). Unlike Rae et al. [8], we did not have an extra washout period nor second baseline testing after the first supplementation. Instead, we relied on the 6 weeks of placebo supplementation for washing out the creatine. The trial evaluated cognitive performance after creatine compared to placebo. The trial design and participant flow are summarised in Fig. 1. We follow the CONSORT reporting guidelines [30].

### Participants

Participants were 18 years or older (see Appendix for full list of inclusion criteria). Half of them reported being on a vegetarian diet and half of them on an omnivore diet. A screening questionnaire assessed if the eligibility criteria were met. Participants who met these criteria went through the baseline assessment and were given their first supplement to take home (or for participants tested online, received the two supplements via the mail). Cognitive assessments of participants took place in the clinic laboratory. Due to the contact restrictions due to the COVID-19 pandemic, after 04/2020, participants were tested online via video call instead.

### Interventions and similarity of treatment groups

Participants took the supplements daily for 6 weeks, including the day of the testing. The creatine supplement consisted of creatine monohydrate powder “CreaPure PG” produced by the company Alzchem (Trostberg, Germany). The placebo supplement consisted of maltodextrin powder “Maltodextrin 6” produced by the company Nutricia (Frankfurt am Main, Germany).

The cans looked exactly the same except for clear markings of which one was the first and which one the second supplement. The two powders looked exactly the same and were flavourless. The solubility was somewhat different: While the placebo powder was completely soluble in water and did not settle, the creatine powder slowly settled. We were initially not aware of this difference in solubility. After we noticed it (after the first 40 participants), we asked participants to stir the powder into yoghurt or food with a similar consistency, as we had found no perceptible difference then. To check to what extent blinding was achieved, directly after the last testing, participants were asked to guess what their first supplement had been.

### Outcomes

We had two primary outcomes:

- A standardised 10-min subtest of Raven Advanced Progressive Matrices (RAPM) [8]
- The Wechsler auditory Backward Digit Span (BDS) [31]

RAPM is a test of abstract reasoning. Each item in the test consists of a 3 × 3 matrix with pictures of geometric forms. One of the pictures is missing and the task consists of choosing the right picture to fill this gap out of eight alternatives. The full RAPM consists of 80 items and has a time limit of 40 min. We used the same standardised 10-min subtests of the RAPM as Rae et al. [8], consisting of 20 items each. The subtests are constructed to have equal levels of difficulty based on the published normative performance data and Rae et al. [8] additionally verified this in an independent sample (*N* = 20). The RAPM score consists of the sum of correct responses.

The Backward Digit Span is a test of working memory. The tester reads increasingly longer series of digits to the participant whose task it is to remember and repeat them in reverse order. The task starts with two digits. Each length has two series of digits. The test ends after wrong answers to two series of the same length. The BDS score consists of the sum of correct responses.

We had eight further exploratory outcomes:

- The D2 Test of Attention [32], a test of sustained attention
- The Trail-Making-Test A (TMT-A), a test of visual attention [33]
- The Trail-Making-Test B (TMT-B), a test of task switching [33]
- The Block-Tapping-Test, a test of visuospatial working memory [34]
- The Auditory Verbal Learning Test (AVLT, in German: VLMT), a word-learning test including immediate recall, delayed recall, and recognition [35]
- The Brief-Visuospatial-Memory Test—Revised (BVMT-R), a test of visuospatial memory [36]
- The Stroop test (in German: Farb-Wort-Interferenz Test, a test of inhibitory control [37]
- Regensburger Wortflüssigkeitstest, a test of verbal fluency [38]

Participants reported side effects experienced during the supplementation period in a free text form on the day of testing. How side effects would be grouped for the report was determined after evaluating all entries. At baseline testing, participants performed a test of crystallised intelligence called “Mehrfachwahl-Wortschatztest (MWT-B)” [39]. In this test, participants had to identify real German words among made-up words.

### Sample size

The sample size of 123 was powered (with power = 0.8, alpha = 0.05, calculated with GPower [40]) to detect the effects of Cohen’s *d* = 0.45. The sample size (preregistered as 120) was chosen based on a conservative estimate (see Appendix) of the effect size in Rae et al. [8] (*d* = 1 for both RAPM and BDS) with a substantial buffer to account for smaller effects.

Block-tapping was originally performed with physical blocks and later on the website Psytoolkit [41, 42] as part of remote testing during the COVID-19 pandemic. Because the remote version was not immediately available, the participant number is lower for this task.

### Randomisation and blinding

The order of the two supplements was randomised with Excel by the pharmacy of the University Hospital Heidelberg. They labelled each of the cans of supplements with the participant code and “A” or “B”, corresponding to the first and second supplement. The staff members who tested participants also provided the participants with the supplement cans. Allocation concealment was performed using sequentially numbered, opaque sealed envelopes (SNOSE). Participants and all staff who interacted with them were kept blinded to the allocation (also see intervention section).

### Statistical methods

For each cognitive test, we conducted a mixed ANOVA with test score after supplementation as the dependent variable, supplement (creatine vs placebo) as the within-subjects factor and supplement order (creatine-first vs placebo-first) as the between-subjects factor^1^. We did not remove outliers in our main analysis, but conducted robustness checks which included trimming and winsorising. We applied the Greenhouse–Geisser correction to all our analyses but the correction did not change any value. We did not exclude participants based on adherence (intention-to-treat analysis). We analysed participants’ actual and not assigned order of supplements. Actual and assigned order differed for only one participant.

We conducted a frequentist analysis, because it is widely understood and it offers the insight of how likely the data is under the null hypothesis. However, we also wanted to know whether the data was more likely under the null or under the alternative hypotheses, i.e. in which direction to update our credence, and to what extent. We conducted a Bayesian analysis, because it answers this question. We were interested in comparing the null hypothesis to different alternative hypotheses—postulating a large effect like in the study we replicated [8] and postulating effects of smaller, in our view more realistic, sizes. A significant result does not imply Bayesian evidence in favour of the alternative hypothesis, nor does a nonsignificant result imply Bayesian evidence against the alternative hypothesis [43]. If the alternative hypothesis postulates a relatively large effect, the data may be more likely under the null than the alternative hypothesis, despite a *p*-value below 0.05. If the alternative hypothesis postulates a relatively small effect, the data may be more likely under the alternative than the null hypothesis, despite a *p*-value above 0.05.

### Confirmatory analyses

As preregistered, our two confirmatory cognitive tasks are the Backward Digit Span and Raven’s Advanced Progressive Matrices. All other cognitive tasks are analysed in an exploratory fashion. There is one deviation from our preregistered analyses. We had preregistered *t*-tests, but this was a mistake in the preregistration. The *t*-test is not appropriate here because imbalances in the supplement order group sizes would bias the results. Instead, we conducted mixed ANOVAs with supplement (creatine vs placebo) as the within-subjects variable, supplement order (creatine-first vs placebo-first) and diet (vegetarian vs omnivore) as the between-subjects variables, and test score after supplementation as the dependent variable.

### Robustness checks

We checked the robustness of our normality-assuming ANOVAs by performing: an ANOVA on 20%-trimmed data, an ANOVA on 5%- and 20%-winsorised data, and a robust ANOVA which uses trimming and bootstrapping (performed with the sppb functions in the WRS2 R package). The latter ANOVA provides the most robust estimate of these methods [44, 45].

### Bayes factors

For the calculation of the Bayes factors, we used the estimated marginal means (EMMs) of the creatine and placebo score. The EMMs are the means weighed for the order groups (creatine-first and placebo-first), so that imbalances in the sizes of the order groups do not affect the means. So, we only had two groups for the Bayes factor calculation (creatine and placebo), simplifying the analysis. The mean difference and standard error of the mean difference were used to describe the data. Using the Bayesplay package [46], we calculated the Bayes factors in several different ways. Approach 1 used point models for the null hypothesis and the alternative hypotheses. Approach 2 compared a point null model against half normal distributions centred on zero and with the standard deviation set to half the maximum expected effect size. For the reasons behind this, see the Appendix.

### Exploratory analyses

In addition to the confirmatory analyses of BDS and RAPM, we analysed the other cognitive tasks in the same way in an exploratory fashion.

We also looked in an exploratory fashion at the first supplementation and the second supplementation separately and at participants with a low and high baseline performance separately (see Appendix).

## Results

### Participant flow

See participant flow in Fig. 1.

Drop-outs were due to supplements failing to arrive (*N* = 2), dealing with stressful personal events (*N* = 2), no reason given (*N* = 2), and no time (*N* = 18).

### Recruitment

Participants were recruited through flyers and social media between 05/2019 and 05/2022 and tested between 05/2019 and 08/2022.

### Baseline data

We analysed all available participant data apart from two minor exceptions (see Appendix). Participants were included irrespective of their adherence. The median number of days per week with meat consumption for omnivore participants was 3.5 (mean = 3.7, SD = 2.0). For further participant characteristics, see Table 1.

**Table 1:** Participant baseline characteristics

*N*	Total	Creatine-first	Placebo-first
	125	63	62
Age in years (M, SD)	30.6 (10.1)	31.5 (10.4)	29.8 (9.7)
Sex (% female)	57%	54%	60%
Weight in kg (M, SD)	70.3 (13.7)	71.8 (15.5)	68.8 (11.4)
MWT-B (M, SD)	26.31 (4.35)	26.32 (3.99)	26.31 (4.72)

Data is given as mean (standard deviation) or as percentage. The MWT-B (Mehrfach-Wahl-Wortschatztest) is a test of crystallised intelligence [39]

### Blinding, adherence, and side effects

After their final testing session, the last 73 participants were asked to guess the order of their supplements (as the idea did not occur to us before). Forty-three (59%) guessed correctly and 30 (41%) guessed incorrectly. A binomial test reveals that the probability of 43 or more correct guesses out of 73 by pure chance is *p* = 0.080. However, most participants who guessed correctly reported being very unsure about their guess. We recorded the reasons for the guesses of the last of the 33 participants. Of those participants who had a reason for their guess, solubility was the most common, followed by negative side effects and positive side effects. All three reasons seemed to improve guess accuracy (see Appendix).

A *z*-score test for two population proportions revealed that the proportion of participants reporting any negative side effect was significantly higher for the creatine than the placebo condition, *p* = 0.002, RR = 4.25 (Table 2). In addition, although we did not assess this systematically, some participants reported positive side effects such as improvements in strength (several participants) and mood (one participant). No patients discontinued the study due to an adverse event.

**Table 2:** Adherence and negative side effects

	Creatine	Placebo
Days supplemented per week (M, SD)	6.89 (0.26)	6.87 (0.26)
Any side effects	17%	4%
Of these		
Digestion problems	6%	2%
Weight gain	3%	0%
Other		
- Tiredness	1x	1x
- Thirst	1x	1x
- Weight loss	1x	0x
- Nightmares	1x	0x
- Cramps	1x	0x
- Thoughts racing	1x	0x
- Problems concentrating	1x	0x
- Nervousness	1x	0x

Adherence (self-reported) was high (Table 2). All but one participant took the supplements in the order assigned to them. This participant was analysed with their actual, not their assigned, supplement order.

### Interaction with diet

There was no significant interaction between diet and supplement nor between diet, supplement, and supplement order for neither BDS (*p* = 0.808 and *p* = 0.559) nor RAPM (*p* = 0.392 and* p* = 0.606), nor was the interaction in the predicted direction (we had hypothesised that vegetarian participants would benefit more from creatine than omnivore participants). This was also true when using the robust ANOVA based on bootstrapping. Bayes factors favoured the null hypothesis. To be precise: They indicated strong support in favour of the null hypothesis over the effect size in Benton and Donohoe [23] (*d* = 0.36) and weak to strong support in favour of the null hypothesis over smaller effect sizes (see Appendix). There was no indication for an effect of diet in the exploratory cognitive tasks either. For more details on the analysis of diet, see the Appendix.

### Confirmatory analysis

There was a significant interaction between supplement and supplement order for both BDS and RAPM. This seems to reflect a learning effect (see Appendix). The effect of most interest, the main effect of the supplement, was in the expected direction but not significant. However, it bordered on significance for BDS (*p* = 0.067, *η*^2^_P_ = 0.028). This means that 2.8% of the variance in BDS scores that was not already explained by other variables was explained by the supplement. For RAPM, it was 0.9%. The supplement effect was virtually the same whether diet was included as a variable or not (Table 3). Thus, we simplified additional analyses (estimated marginal means, Bayes factors, and robustness checks) by dropping diet as a variable for these analyses.

**Table 3:** Results of confirmatory analysis

Task	*N*			**Supplement effect** **3-way ANOVA, inkl. diet**			**Supplement effect** **2-way ANOVA**			Crea. score	Pl. score	Crea.-Pl. scores M(SE) [95% CI]
	Total	Crea. first	Pl. first	*F* (df)	***p***	***η***^**2**^_**P**_	*F* (df)	***p***	***η***^**2**^_**P**_			
BDS	121	61	60	3.41 (1, 117)	**0.067**	**0.028**	3.49 (1, 119)	**0.064**	**0.029**	8.85 (0.28)	8.44 (0.25)	0.41 (0.22) [–0.24; 0.84]
RAPM	118	60	58	1.02 (1, 114)	**0.315**	**0.009**	0.97 (1, 116)	**0.327**	**0.008**	12.39 (0.28)	12.16 (0.28)	0.23(0.23) [− 0.24; 0.70]

Mixed 3-way ANOVA with supplement (creatine vs placebo) as the within-subjects variable, supplement order (creatine-first vs placebo-first) and diet (vegetarian vs omnivore) as the between-subjects variable and test score after supplementation as the dependent variable. Mixed 2-way ANOVA without diet. The test score is given as estimated marginal mean (standard error). *P*-values are two-tailed. The two cognitive tasks are the Backward Digit Span and Raven’s Advanced Progressive Matrices

**Fig. 2:**
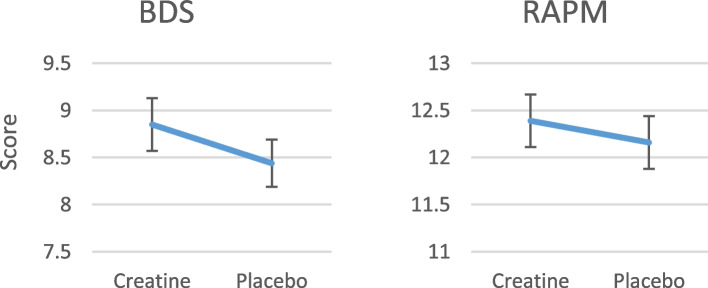
**a** Estimated marginal means for the Backward Digit Span (BDS) score. **b** Estimated marginal means for Raven’s Advanced Progressive Matrices (RAPM) score. Error bars represent standard errors

In terms of raw scores, the effect size for BDS was 0.41 additional correct items, i.e. a 0.2-digit longer digit span, because there were always two-digit spans of the same length. For RAPM, the effect was 0.23 more matrices solved (Fig. 2). If these were IQ tests, this increase in raw scores would mean 2.5 IQ points for BDS (using the standard deviations of a normative study [47] or our own baseline gives the same result, see Appendix). For RAPM, the improvement would be 1 IQ point (using the standard deviation of our own baseline, see Appendix). Cohen’s *d* based on the estimated marginal means of the creatine and placebo scores was 0.09 for RAPM and 0.17 for BDS.

### Bayes factors

To facilitate the interpretation of the results of the confirmatory analysis, we provide Bayes factors. A Bayes factor (BF_10_) indicates how likely a null hypothesis is compared to an alternative hypothesis given the data. A BF_10_ between 1/3 and 3 indicates low sensitivity of the data (i.e. not enough data to be certain), with weak evidence in favour of the null hypothesis if BF_10_ is below 1 and weak evidence in favour of the alternative hypothesis if it is above 1. A BF_10_ above 3 (below 1/3) is considered moderate and above 10 (below 1/10) strong evidence [48].

We compare several alternative hypotheses postulating small beneficial effects of creatine to the null hypothesis. For RAPM, the data was very insensitive, very weakly favouring the alternative hypotheses. For BDS, the data was more sensitive, providing weak to moderate support in favour of the alternative hypotheses. Two different approaches to calculating these Bayes factors were used (see statistical analysis) and the results were similar (Table 4).

**Table 4:** Results of Bayesian analysis

**Task**	**Approach 1: point models**				**Approach 2: half normal**		
	Small effects			Rae-sized	Small effects		Max. = 2 × Rae-size
	**0.1**	**0.2**	**0.4**	**2.5**	**max. 0.4**	**max. 1**	**max. 5**
BDS	2.1	3.6	5.7	< 2e − 7	2.9	3.3	1.0
RAPM	1.4	1.6	1.3	< 2e − 7	1.4	1	0.3

Bayes factors (BF_10_) comparing a range of alternative hypotheses to the null hypothesis. The effect size is given as the raw score difference. Approach 1 compared a point null model to point alternative models with a range of small effect sizes (0.1–0.4, i.e. *d* = 0.04–0.17) as well as an equivalent of Rae et al.’s effect size (2.5, i.e. *d* = 1, see calculation in Appendix). Approach 2 compared a point null model against half-normal distributions centred on zero and with the SD set to half the maximum expected effect size

There was strong evidence in favour of the null hypothesis compared to the alternative hypothesis postulating the effect size found by Rae et al. [8]. The data was insensitive (BDS) or weakly favoured the null hypothesis (RAPM) when compared to the half-normal model based on Rae et al. [8]. The half-normal model based on Rae et al. [8] does not assume their effect size is the true effect size in the population. Instead, the model assumes their effect size is a moderate overestimation of the true effect size. The model uses their effect size as a reference point to assign probabilities to effect sizes. It assigns most of the probability weight to effect sizes that are smaller than this effect size, and some probability to effect sizes up to twice that effect size. This is a common alternative model when replicating studies. However, we did not use it as our only model, because we were also interested in assessing the likelihood of smaller effect sizes and of the possibility that the effect size in Rae et al. [8] was the true population effect size.

The results were similar whether using normal or Cauchy distributions. For more details on this and the aforementioned calculations, see the Appendix.

In summary, this study provides weak to moderate evidence for a small cognitive benefit of creatine and strong evidence against the effect size by Rae et al. [8] being representative.

### Robustness checks

We checked the robustness of our confirmatory analysis (the normal ANOVA) by performing an ANOVA on 20%-trimmed data, an ANOVA on 5%- and 20%-winsorised data, and an ANOVA which uses bootstrapping and 20% trimming.

For RAPM, all of these methods gave overall similar results to that of the normal ANOVA (Table 5).

**Table 5:** Results of robustness checks

Task	Better score	Max. skew	*p* (Supplement)				
			Normal	20% trim	5% winsorisation	20% winsorisation	**Bootstrap and 20% trim**
RAPM	creatine	− 059	0.327	0.412	0.361	0.672	0.354
BDS	creatine	1.14	0.064	0.17	0.009	0.05	0.37

Creatine effect *p*-values (two-tailed) for different ANOVAs. The given trim and winsorisation percentages are applied to each side. Better score based on estimated marginal means. “Max. skew” gives the highest skewness statistic in any combination of conditions (supplement and supplement order)

For BDS, whose skewness statistic was slightly further from 0 than that of RAPM, these methods gave results that differ from each other and from the normal ANOVA to a relevant extent (Table 5). Most notably, the *p*-value for the supplement effect was 0.009 for the 5% winsorisation and 0.370 for the bootstrap ANOVA. This seems to suggest that in the normal ANOVA, the most extreme values made the effect of creatine appear smaller by inflating the variance, while relying on possibly unjustified assumptions of normality made the effect of creatine appear larger.

Thus, the result for RAPM was robust and for BDS much less so.

### Exploratory cognitive tasks

There was no indication that creatine improved the performance of our exploratory cognitive tasks. The distribution of *p*-values was what one would expect if there was no effect. For the exploratory cognitive tasks, Table 6 only includes the *p*-values of the supplement effect. For the full results, including the interaction effect (reflecting a learning effect) and the order of supplement effect, see the Appendix.

**Table 6:** Results for exploratory cognitive tasks

Task	*N*	Better score	*p* (Supplement)				
			Normal	20% trim	5% winsorisation	20% winsorisation	**Bootstrap and 20% trim**
Block-tapping forward	71	creatine	0.779	0.564	0.865	0.678	0.826
Block-tapping backward	70	placebo	0.83	0.87	0.482	0.482	0.59
BVMT-R	119	creatine	0.543	0.809	0.746	0.112	0.67
D2 test	104	placebo	0.394	0.382	0.446	0.291	0.62
Forward digit span	117	placebo	0.714	0.795	0.838	0.721	0.52
Stroop—colors	118	placebo	0.813	0.184	0.432	0.054	0.568
Stroop—colorletters	119	placebo	0.626	0.877	0.861	0.547	0.856
TMT A	123	placebo	0.129	0.04	0.068	0.021	0.224
TMT B	122	creatine	0.855	0.622	0.745	0.567	0.56
VLMT immediate recall	119	creatine	0.87	0.744	0.996	0.883	0.996
VLMT recall after interference	119	creatine	0.694	0.622	0.854	0.323	0.806
VLMT delayed recall	118	placebo	0.339	0.346	0.462	0.133	0.54
VLMT recognition	117	creatine	0.722	0.327	0.398	0.635	0.348
Word fluency	122	creatine	0.227	0.631	0.272	0.119	0.692

Creatine effect *p*-values (two-tailed) for different ANOVAs. The given trim and winsorisation percentages are applied to each side. Higher score based on estimated marginal means

## Discussion

### Summary of results

This is the largest study on the cognitive effects of creatine to date. As part of our study, we aimed to replicate Rae et al. [8], who found a large positive effect of creatine on the abstract reasoning task Raven’s Advanced Progressive Matrices (RAPM) and on the working memory task Backward Digit Span (BDS) in healthy young adult vegetarians.

We found Bayesian evidence for a small beneficial effect of creatine on cognition for both tasks. Cohen’s* d* based on the estimated marginal means of the creatine and placebo scores was 0.09 for RAPM and 0.17 for BDS. If these were IQ tests, the increase in raw scores would mean 1 and 2.5 IQ points. The preregistered frequentist analysis of RAPM and BDS found no significant effect at* p* < 0.05 (two-tailed), although the effect bordered significance for BDS. There was no influence of diet (vegetarian vs omnivore), age, or sex on this effect. There was no indication that there was a creatine effect in several other cognitive tasks that we studied in an exploratory analysis.

### Effect of diet

Dietary creatine is primarily contained in meat, fish, and a small amount in some dairy products [13, 14]. So, creatine is almost non-existent in a vegetarian diet. This might lead one to expect vegetarians to benefit more from creatine supplementation than omnivores do. However, even in an omnivore diet, creatine intake through the diet is low compared to common doses for creatine supplementation. Therefore, it is possible that the low dose in the diet does not affect cognition, while the higher dose of supplementation does. As opposed to muscles, which always receive creatine from other parts of the body, the brain synthesises its own creatine and for that reason might be more resistant to exogenous creatine [22, 49]. This might mean that higher doses of creatine are necessary to increase brain creatine levels [22, 50, 51]. In our study, half of the participants were vegetarians and half of them were omnivores. We found no indication that our vegetarian participants benefited more from creatine than our omnivore participants (in fact, the creatine effect was smaller in vegetarians than omnivores to a non-statistically significant extent). This is in line with Solis et al. [22, 24] who did not find a difference in brain creatine content between omnivores and vegetarians. Our Bayesian analysis of their data provides moderate support for the lack of a difference (see Appendix). In contrast, Benton and Donohoe [23] found that creatine supplementation benefited memory in vegetarians more than in omnivores, with no difference in baseline performance. However, given the high number of cognitive tasks in that study, the chance of a false positive was high, so we regard their finding as exploratory. Apart from the present study and Benton and Donohoe [23], we are not aware of any other RCT comparing the effect of creatine supplementation on cognition between vegetarians and omnivores.

Rae et al. [8] is the only RCT on the effect of creatine supplementation on cognition with only vegetarian participants (healthy, under normal conditions) and they found a large creatine effect. Under the same conditions, a number of other studies with omnivore participants [52–55] or unspecified and presumably omnivore participants [56–58] also found a large creatine effect, while other studies with omnivore participants [21, 59, 60] or unspecified and presumably omnivore participants [61] failed to find a creatine effect^2^^,^^3^. The omnivore findings are mixed while the only study on vegetarians [8] is positive, but as it is only one study, it is not a strong indication.

Observational data on the role of dietary creatine in cognition is conflicting. Ostojic et al. [62] (1340 elderly adults in the USA) found a significant positive correlation between performance in the Digit Symbol Substitution Test and amount of meat consumed. By contrast, two studies by Giem et al. [63] in Seventh Day Adventist adults in the USA (272 participants matched for age, sex, and zip code and 2984 unmatched participants) both found a trend towards delayed onset of dementia in vegetarians compared to omnivores. Many confounders are possible in these observational studies, such as health influencing both diet and cognitive performance, differences in lifestyle, and components in meat other than creatine. None of the studies assessed creatine supplementation.

Overall, observational evidence is mixed. Evidence from RCTs and brain creatine studies does not support the idea that dietary creatine affects brain health.

### BDS and other short-term memory tasks

While the creatine effect for BDS in our study bordered significance, it was smaller (*d* = 0.17) compared to the two other studies in the literature that have tested the effect of creatine supplementation on BDS in young, healthy adults (Rae et al. [8]; Hammett et al. [56])^4^.

One reason for this could be that there might have been more noise in our study, maybe due to the COVID pandemic starting in the midst of the study. This reason might apply to all of our tests. However, the standard deviation for BDS in this study was almost exactly the same as in the study we aimed to replicate^5^ [8] (2.42 vs 2.38) and our sample size was much larger, so noise does not explain the difference in results. Our supplementation protocol was different from that of Hammett et al. [56], which could lead to different results. However, the other study [8] used the same supplementation protocol as ours (5 g/day for 6 weeks), so this reason seems unlikely. Compared to Rae et al. [8], one might at first glance think that their effect was larger because all of their participants were vegetarians whereas only half of our participants were vegetarians. However, the number of vegetarian participants in our study was still higher than theirs and the creatine effect was not larger in this subgroup (in fact, it was smaller to a non-statistically significant extent). There are reasons to think that creatine supplementation becomes more beneficial with age (more on this below). However, the age of our participants was very similar (mean = 31, sd = 10, median = 28) but slightly higher than those of Rae et al. [8] (median = 25.5) and Hammett et al. [56] (median = 26), so age does not seem to explain the difference in results. Overall, we did not find a convincing reason for why our BDS result is different from the two other studies with young, healthy adults. The difference is not explained by diet, age, or noise.

Three further studies tested the effect of creatine supplementation on BDS in a different population than healthy young adults. In two of these studies, participants were healthy and elderly [55, 61] and in one study sleep-deprived [64]. In these three studies, there was no creatine effect. This is surprising, seeing as theory and evidence generally suggest that sleep-deprived and older participants have a larger cognitive benefit from creatine supplementation (more on this below). One of the two studies on healthy elderly participants, Alves et al. [61], also tested the effect of creatine on strength and found no effect, which goes against a large body of literature. The effect of creatine supplementation on strength is well-studied and generally large. Given that this effect was not present in Alves et al. [61] makes it less surprising that they did not find an effect for cognition. The results of the two studies by McMorris et al. are more difficult to make sense of. The creatine effects for BDS in these studies were smaller than in the present study, while the effects in studies with the same kind of population as ours were larger than in the present study. Ignoring the differences in populations for a moment, it might simply be that the real effect of creatine on BDS performance lies in the middle like our study.

In a review by Avgerinos et al. [7] on the effects of creatine supplementation on cognition, studies testing BDS were grouped together with two studies with other short-term memory tasks: Benton and Donohoe [23] and Rawson et al. [60]. The short-term memory task in Benton and Donohoe [23] consisted of the recall of 30 words. There was a creatine effect only for their vegetarian participants for this task. However, as mentioned in the discussion section on diet, this study included a large number of tasks, so we consider this finding exploratory. The short-term memory task in Rawson et al. [60] was the Sternberg task, where participants had to memorise 6 letters displayed for 20 s and subsequently decide if “probe” letters were included or not in those 6 letters. Rawson et al. [60] found no effect of creatine, which Avgerinos et al. [7] attributed to the participants’ young age (M = 20, SD = 2.2). While this is plausible in theory and there is evidence supporting older people benefiting more from creatine, it is unclear to what extent it explains the differences in findings in this case, as two studies with participants who were only slightly older [8, 56] found large creatine effects for short-term memory while the only two studies with elderly participants found no effect.

Five of our exploratory tasks, the forward digit span, forwards and backwards block-tapping task (spatial), the BVMT-R, and the immediate recall part of the VLMT also tested short-term memory and there was no indication of an effect for these tasks. A review by Dolan et al. [50] reports that a creatine effect is more likely for more cognitively demanding tasks. In line with this, we found some indication of a creatine effect for the backward digit span (BDS) but not for the less demanding forwards digit span and block-tapping tasks. The VLMT and BVMT-R may also be less cognitively demanding than the BDS, but this comparison is less obvious to make. Going against this idea, McMorris et al. [55] found a creatine effect for short-term memory tasks that we deem less demanding but not for BDS.

Overall, the effect size for BDS in the present study lies in between that of other studies, some of which found very large effects and some of which found no effect. The reason why we found an effect for BDS, which is a short-term memory task, but not for our exploratory short-term memory tasks, might be that BDS is probably more difficult and thus requires a higher ATP turnover, which is where creatine helps. Short-term memory is critical for language comprehension, learning, planning, reasoning, and general fluid intelligence [65], so even a small improvement in the most difficult tasks might be very valuable.

### RAPM and other abstract reasoning tasks

In our study, the creatine effect for Raven’s Advanced Progressive Matrices (RAPM) was much smaller (*d* = 0.09) compared to two of the three other studies in the literature that have tested the effect of creatine supplementation on RAPM in healthy adults [8, 54, 56]. It is unclear how our effect for RAPM compares to that of the third study [56] because they did not report this effect size or any other information that could be used to calculate it. However, as their creatine effect for RAPM was in the predicted direction but not significant and presumably also not bordering significance, the effect in that study was likely not very different from that of the present study. So, our results are likely in line with those of Hammett et al. [56]. Hammett et al. [56] used a shorter version of the RAPM (5 min) compared to Rae et al. [8] (10 min). It seems plausible that this might have made the task less robust and thereby reduced the effect.

Rae et al. [8] have already been discussed above for BDS—the same points apply here. In short: No reason is evident why the effect would be smaller in the present study. Noise in RAPM was lower in the present study than in Rae et al. [8], so this does not explain the result. Again, age does not explain the difference in results. In line with the idea that cognition in young people benefits less from creatine, a large study with children (aged 10–12) did not find a significant creatine effect on RAPM [21]. However, the age of our participants (mean = 31, sd = 10, median = 28) and Hammett et al. [56] (median = 26) was slightly to moderately higher than in the other two adult studies (Rae et al.: median = 25.5; Ling et al.: mean = 21).

In a review by Avgerinos et al. [7], studies testing RAPM were grouped together with a study by Rawson et al. [60] using a logical reasoning task and a mathematical processing task, which did not find a creatine effect. Avgerinos et al. [7] explained this difference in findings with the participants’ age. The participants in Rawson et al. were younger than those of the other studies in the review [8, 54]. However, compared to Ling et al., the age difference is so small (mean age of 20 (SD = 2.2) vs 21 (SD = 1.4) years) that we do not think it explains the difference in the results of the two studies. A reason we find more compelling is the difference between the tasks. The mathematical processing task requires participants to decide if a three-step equation involving addition/subtraction gives a result lesser or greater than five. The logical reasoning task consists of a series of symbols, for example #@, followed by two statements about their order, for example # before @ and @ after #. The task is to determine if both or only one of the statements is consistent with the series. Both tasks seem to differ from RAPM in being easier to solve, less varied and complex, requiring less creativity and providing no progression in difficulty. Alternatively, the negative finding by Rawson et al. could also be explained by the creatine effect in the reasoning domain being smaller than suggested by the findings of Rae et al. and Ling et al. The present study and Hammett et al. [56] are in line with this explanation.

In sum, two studies found much larger creatine effects for RAPM than that in the present study, while a third study did not find a significant effect. Possibly the real effect lies in the middle like our study. RAPM, while not a pure measure of g [66], substantially correlates with general intelligence [66] and predicts academic achievement [67]. Even small improvements would be very valuable.

### Exploratory cognitive tasks other than short-term memory

We did not find a creatine effect for our exploratory tasks. Our negative finding for the verbal fluency task is in line with the only other study using the same task [23]. Our negative finding for the long-term memory part of the VLMT is in line with two studies assessing long-term memory [21, 61] and in contrast with one study which found a creatine effect for this domain [55]. The study which found an effect on long-term memory used an idiosyncratic task in which participants had to remember a combination of occupations and photographs of faces. It might be that this task has characteristics that make it more susceptible to benefit from creatine—e.g. perhaps it is more difficult. Our negative finding for the trail-making task, which tests task switching, is in line with the same two studies [21, 61]. Our negative finding for the Stroop task, a test of inhibition, is again in line with the same two studies [21, 61] and in contrast with one study which found a creatine effect for this task [57]. In contrast to Van Cutsem et al. [57], Alves et al. [61] found no creatine effect on strength and the participants in Merege-Filho et al. [21] were children (aged 10–12). These differences might explain why Van Cutsem et al. [57] found an effect while the other two studies did not find an effect of creatine for any of their tasks. In addition, Van Cutsem et al. [57] modified the Stroop task in various ways that increased its difficulty. More difficult tasks have been hypothesised to benefit more from creatine supplementation [50], because they require more energy, i.e. a higher ATP turnover, which is benefited by creatine. This modification might be why Van Cutsem et al. [57] found a creatine effect for this task while the present study did not.

In sum, for the exploratory tasks, overall the evidence does not support a creatine effect. However, as the evidence for the Stroop task shows, this might be only when the tasks are made too easy for participants, so that creatine has no chance to help.

### Effect of age

It has been claimed that creatine supplementation is more likely to benefit older adults more than younger ones [7, 68, 69]. So, one reason why creatine did not impact most of the cognitive tasks in this study might be that most of our participants were relatively young (mean = 31, sd = 10, median = 28).

One theory behind an effect of age is that brain creatine levels might decrease with age. There is evidence that this happens with muscle creatine levels [70–73] (although see [74, 75]), although it is unclear if this is an effect of ageing itself or a result of other reasons such as dietary choices or reduced physical activity [22]. Similarly, brain creatine might be affected by ageing directly or mediated by reduced brain activity. However, Solis et al. [22] found no difference between the brain creatine levels (nor muscle creatine) of young and elderly adults, which speaks against this theory.

Experimental evidence suggests an effect of age. A meta-analysis by Prokopidis et al. [68, 69] on RCTs with healthy participants on an omnivore diet found that creatine supplementation significantly improved memory in older adults (aged 66–76 years) but not younger adults. However, there are reasons for caution. The meta-analysis included only two studies (*N* = 25 and *N* = 32) with elderly participants [55, 61], only one of which found a creatine effect. For reasons unknown to us, the meta-analysis did not include Hammett et al. [56], who found a large creatine effect on memory in young adults and might have changed the conclusion. In addition, as described above, a number of other studies with participants younger than ours found large creatine effects. The observational evidence reported in the discussion of diet [62] is consistent with an effect of age. However, as they did not include young adults, we cannot know if they would not show the same correlation.

### Effect of gender

In their review, Smith-Ryan et al. [76] theorise that women might benefit more from creatine supplementation than men. The limited evidence from the present study and previous studies does not support this idea.

There have only been three RCTs on the effect of creatine on cognition in healthy women and three in healthy men. One of the studies with women found a creatine effect and two did not find an effect (one of these with only elderly participants). Two of the studies with men (one of these with sleep-deprived participants) found a creatine effect and one did not find an effect. Apart from the present study, which found no effect of sex, studies who included both men and women did not report the effect of sex. In their meta-analysis on the effect of creatine on memory performance, Prokopidis et al. [68, 69] found no effect of sex.

### Limitations

There are a number of limitations to this study. Despite the large sample size compared to other studies, a larger sample size would be needed to be powered for effects that are smaller but still relevant. Some of the data (4%) could not be analysed because it was not labelled with the participant and timepoint. The COVID-19 pandemic started in the middle of the study, might have added noise to the data, and meant that we had to switch from in-person cognitive testing to testing via video call. However, we do not see this potential source of noise reflected in the standard deviations compared to pre-pandemic studies. We assessed baseline days per week of meat consumption (median = 3.5, mean = 3.7, SD = 2.0), but not grammes per day of consumption. However, creatine intake through meat is usually substantially lower than the supplemented dose [13]. Adherence was self-reported and not checked with blood samples. A major limitation is that brain creatine levels were not assessed. Another limitation is that the proportion of participants who correctly guessed their supplement order (59%) bordered on significance (*p* = 0.08). However, most participants who guessed correctly reported being very unsure about their guess. The largest contributing factor to correct guesses was likely the difference in the solubility between the powders, followed by negative and positive side effects. We attempted to counteract differences in solubility by recommending participants to stir the supplements into yoghurt. Mixing creatine in yoghurt rather than water might have negatively affected creatine absorption, because the lower water content and usually cold temperature of yoghurt would decrease creatine solubility [77]. However, yoghurt is still relatively liquid and has a high water content of about 70–90% [78]. Yoghurt provides carbohydrates (4–18 g/100 g) [78], which has been found to aid creatine retention [27, 79]. In addition, yoghurt has a lower pH value than water (4 vs. 6.5–9-5) [80, 81], a factor likely aiding absorption [82]. Overall, our educated guess is that creatine absorption when mixed in yoghurt is similarly effective as in cold water but worse than in warm water. For future studies, we recommend cellulose as the placebo and a mixture of cellulose and creatine as the treatment, as these two look extremely similar when dissolved in water. The alternative solution with capsules would require participants to consume many capsules per day. This would likely reduce adherence and massively increase costs. Unfortunately, it is difficult to achieve perfect blinding when side effects occur with higher frequency in the creatine condition. The side effects of creatine are well-known and not dangerous [1, 4–6].

## Conclusions

Supplementing creatine is safe, easy, and very cheap. The real effect of creatine on cognition is likely smaller than that reported in Rae et al. [8]. However, even small improvements in cognition may be relevant, especially if accumulated over many people and over time. The results of this study do not allow any strong conclusions, but it would be worthwhile to test for a small effect of creatine in strategically designed, larger studies.

### Supplementary Information

**Additional file 1.**

## Data Availability

The appendix, protocol, data, code, and output of this study are openly available at the Open Science Framework, https://osf.io/xpwkc/.

## Abbreviations

95% CI: 95% Confidence interval
ANOVA: Analysis of variance
ATP: Adenosine triphosphate
AVLT/German: VLMT: Auditory Verbal Learning Test/German: Verbaler Lern- und Merkfähigkeitstest
BDS: Backwards Digit Span
BF_10_: Bayes factor that indicates how likely a null hypothesis is compared to an alternative hypothesis given the data
BVMT-R: Brief Visuospatial Memory Test-Revised
Crea.: Creatine
df: Degrees of freedom
EMM: Estimated marginal mean
*F*: *F*-value
IQ: Intelligence quotient
M: Mean
MWT-B: Mehrfach-Wahl-Wortschatztest, a test of crystallised intelligence
*p*: *p*-value
Pl.: Placebo
RAPM: Raven’s Advanced Progressive Matrices
RCT: Randomised controlled trial
RR: Relative risk
SD: Standard deviation
SE: Standard error
SNOSE: Sequentially numbered, opaque sealed envelopes
TMT-A: Trail-Making-Test, version A
TMT-B: Trail-Making-Test, version B
*η*^2^_P_: Partial eta squared

## Footnotes

1 We used the score after supplementation and not change from baseline, because subtracting the same baseline from both after-supplement scores in a crossover study would cancel out, which would give the same result but complicate the analysis unnecessarily. The baseline was used for describing participants’ baseline characteristics and for exploratory analyses.

2 Samadi et al. [59] mistakenly concluded they had found a creatine effect because their creatine group improved significantly while their placebo group did not. However, the creatine effect is correctly measured by the difference in change between the two groups (i.e. the time × supplement interaction) and this was not significant (*p* = 0.84), nor does it likely give Bayesian evidence in favour of a creatine effect, although we did not check this. Note also that in addition to creatine and placebo, both groups received beta-alanine.

3 One of the studies with omnivores that did not find an effect was on children [48]. Another one also found no effect of creatine on strength, in contrast to a large body of literature [50].

4 Rae et al. [8] was a crossover study like ours. As opposed to us, they included two baselines. To better compare our results to them, we did not just look at their reported effect but also calculated their effect size using just the after-supplement scores like we did in this study (see Appendix). Their effect was still much larger than ours.

5 Using the doubled effect size for Rae et al. [8], because they did not present the same sequence length twice by default like we did.
